# Early diagnosis of hereditary angioedema in children: genetic testing should be prioritized

**DOI:** 10.1186/s13223-025-00950-x

**Published:** 2025-02-11

**Authors:** A. Bocquet, A. Pagnier, I. Boccon-Gibod, F. Defendi, C. Dumestre-Perard, G. Hardy, Laurence Bouillet

**Affiliations:** 1 French national reference center for angioedema (CREAK), Grenoble University Hospital, Grenoble, cedex 09 CS10217 38043 France; 2 Pediatric department, Grenoble University Hospital, Grenoble, France; 3 Immunology laboratory, Grenoble University Hospital, Grenoble, France; 4 Molecular Biology laboratory, Grenoble University Hospital, Grenoble, France

**Keywords:** Hereditary angioedema, C1 INH activity, Genetic research, Early diagnosis

## Abstract

**Background:**

When a member of a family has been diagnosed with hereditary angioedema (HAE) before a child is born, the question of early diagnosis arises. Indeed, the first attacks may occur at birth. Early diagnosis is complicated by biological issues. Due to the immaturity of the complement system, C1 Inhibitor (C1 INH) and C4 levels can be low at birth, generally in the range of 60 to 100% of adult reference values. Like most complement proteins, their levels generally normalize after one year of life. However, this is not always the case, and we report two counter-examples here.

**Case presentation:**

A woman with well-documented HAE due to type II C1 INH deficiency gave birth to two children 4 years apart. Functional C1 INH assays performed at 8 and 7 months of age returned normal C1 INH inhibitory activity. However, a genetic exploration revealed the presence of the mother’s pathogenic gene variant in both children. Subsequent monitoring of C1 INH activity at 3 and 4 years of age confirmed a pathological reduction in C1 INH inhibitory activity.

**Conclusion:**

For the early detection of HAE in children, these cases lead us to recommend genetic testing for the index parent’s pathological variant rather than reliance on results of C1 INH assays.

## Background

The latest international WAO/EAACI guidelines for the management of hereditary angioedema (HAE) recommend biological screening of patients, reserving genetic diagnosis for complex cases only [1]. When a member of a family has been diagnosed with HAE before the birth of a child, the question of early diagnosis arises. Indeed, the first attacks may occur at or even before birth. Due to the young age of the patients, these attacks can take misleading forms with consequent misdiagnosis [2]. For example, newborns may present only diffuse whole-body erythema marginata [3]. Similarly, abdominal attacks tend to be polymorphous in children, opening up the possibility of many differential diagnoses. Finally, but importantly, a newborn may present with laryngeal angioedema, which could be life-threatening. Early diagnosis would also reduce stress for the parents, who will necessarily be worried about having transmitted the disease. The French consensus recommends testing plasma for C1 Inhibitor (C1 INH) function and concentration from the 6th month of life, followed-up with a second assay in the event of a low value. This second assay is important as C1 INH and C4 levels can be low at birth due to the immaturity of the complement system. They are generally in the range of 60 to 100% of adult reference values [4]. Like most complement proteins, levels normalize at around age one year. However, this scenario does not always play out.

Here, we report the cases of two newborns whose initial normal C1 INH activity decreased after age 12 months. Serum protein concentrations of C1 INH and C4 were assayed by nephelometry (Siemens, Marburg, Germany); plasma C1 INH function was assessed based on the residual esterase activity in plasma samples after incubation with the C1s protease, as described by Drouet et al. [5]. Sample collection, preanalytical processing, and storage are also detailed in [5]. Results are expressed as inhibitory activity, i.e., the amount of substrate converted to product per milliliter (U/mL) and as specific activity, i.e., the enzymatic activity per milligram of protein (U/mg).

## Case presentation

The family had a history of HAE linked to type II C1 INH deficiency (HAE-C1INH). Both the mother and the grandmother were affected. The mother had her first attack at 8 years of age. She subsequently experienced more than one attack per month, with peripheral and abdominal localization. The grandmother had her first attack at age 20 years, when she first took a contraceptive pill. She only had peripheral attacks with very few symptoms at a frequency of less than one attack per year. Both women have elevated C1 INH concentration and inhibitory activity below 50% of the reference value (Table 1).

**Table 1:** Complement exploration of HAE-C1INH-type II affected family

	C1 INH concentration (mg/L) RI: 210–355	C1 INH function		C4 concentration (mg/L) RI: 100–380
		Inhibitory activity (U/mL) RI: 17.0-27.4	Specific activity (U/mg) RI:67.4–93.6	
Grand Mother	467	7,6	16,2	-
Mother	334	10,6	31,7	36
Son at the age of				
8 months 4 years	417 403	16.5 12.8	39,6 31.8	105 80
Daughter at the age of				
7 months 3 years	445 443	17.4 6.7	38.5 15.2	92 75

The mother’s first child was screened for complement anomalies at age 8 months: C1 INH and C4 concentrations, as well as C1 INH inhibitory activity were all normal. The specific activity was slightly lower than normal levels. These results falsely reassured the parents. A control assessment at age 4 years returned low C1 INH inhibitory activity (below 60% of the reference value) associated with slightly reduced C4 levels. Molecular analysis, with Sanger sequencing of the gene *SERPING1* (NM_000062.3), identified a well-known heterozygous pathogenic variant at the reactive center in exon 8: c.1397G > A p.(Arg466His). This variant is described as associated with type II HAE-C1INH [6]. To date, the child has not experienced an attack.

The mother subsequently gave birth to a baby girl who was tested for complement deficiency at age 7 months. The C1 INH concentration was normal with inhibitory activity within the reference range (but decreased specific activity). For this child, the genetic investigation was performed at the same time as the biochemical assays. It revealed the presence of the familial pathogenic variant in the heterozygous state. In this patient, complement exploration at age 3 years confirmed low C1 INH inhibitory activity (less than 50% of reference value).

## Discussion and conclusion

In the literature, two comparable cases have been reported by H. Farkas and team [7]. They analyzed samples (cord blood or peripheral blood) from 11 children with C1 INH-related HAE (confirmed by genetic testing). Among their patients, 9 had the expected biological profiles for this disease. However, one of the children had normal C1 INH inhibitory activity levels but reduced C1 INH and C4 concentrations; and a second child (with type II C1 INH HAE) initially had a C1 INH concentration and inhibitory activity in the reference range, but inhibitory activity decreased after one year of age. These cases and ours highlight the value of determining the specific inhibitory activity of C1 INH when testing for type II HAE-C1INH. Indeed, a pathological result alerts to the need for follow-up complement testing.

The evidence from these four cases led us to question the methods used for early detection of HAE-C1INH in France. As a result, CREAK now recommends screening children at birth by genetic exploration, after identification of the causal variant in the affected parent. This test can be carried out on a buccal swab, which is much less traumatic for the newborn than a blood test. For early pre-symptomatic diagnosis without C1 INH assay, confirmation on a second independent sample is recommended.

## Data Availability

No datasets were generated or analysed during the current study.
